# Draft Genome Sequence of *Halomonas* sp. HG01, a Polyhydroxyalkanoate-Accumulating Strain Isolated from Peru

**DOI:** 10.1128/genomeA.01598-15

**Published:** 2016-01-21

**Authors:** Juliana Cardinali-Rezende, Rafael Augusto Teodoro Pereira de Souza Nahat, César Wilber Guzmán Moreno, Carmen Rosa Carreño Farfán, Luiziana Ferreira Silva, Marilda Keico Taciro, José Gregório Cabrera Gomez

**Affiliations:** a Institute of Biomedical Sciences, University of São Paulo, São Paulo, Brazil; b Facultad de Ciencias Biológicas, Universidad Nacional Pedro Ruiz Gallo, Lambayeque, Peru

## Abstract

*Halomonas* sp. strain HG01, isolated from a salt mine in Peru, is a halophilic aerobic heterotrophic bacterium accumulating poly-3-hydroxybutyrate and poly(3-hydroxybutyrate-*co*-3-hydroxyvalerate) from different carbon sources. Here, we report the draft genome sequence of this isolate, which was found to be 3,665,487 bp long, with a G+C content of 68%.

## GENOME ANNOUNCEMENT

The gammaproteobacterium *Halomonas* sp. strain HG01, belonging to the order *Oceanospirillales*, is a moderate halophilic aerobic and heterotrophic bacterium isolated in medium containing 10% NaCl from a salt mine located in San José, Lambayeque, Peru (6°46′8.26″S and 79°56′56.78″W) (1). This strain presented a great potential for poly-3-hydroxybutyrate (P3HB) production from different carbon sources, such as glucose, sucrose, and fructose, under high concentrations of salt. Furthermore, *Halomonas* sp. HG01 presented poly(3-hydroxybutyrate-*co*-3-hydroxyvalerate) (P3HB-*co*-3HV) production when propionic and valeric acids precursors were added to the culture medium (2).

Whole genomic DNA of the strain was extracted using a DNeasy blood and tissue kit (Qiagen, Valencia, CA, USA) and quantified using a Qubit 2.0 fluorometer (Life Technologies, USA). We obtained 550-bp DNA fragments using a Covaris S2 ultrasonicator (Covaris, Inc.), which were subsequently visualized on agarose gel electrophoresis and quantified using a Qubit 2.0 fluorometer. A paired-end sequencing library was constructed using a TruSeq DNA PCR-free LT sample preparation kit (Illumina, San Diego, CA, USA). The library insert size was verified using a 2100 Bioanalyzer (Agilent Technologies, USA) and quantified by real-time PCR (qPCR) using a library quantification kit for the Illumina genome analyzer (Kapa Biosystems, MA). The sequencing library was prepared according to the manufacturer’s protocols and sequenced on an Illumina MiSeq.

A total of 2,743,560 reads were generated, decreasing to 1,414,506 reads after quality control was performed using the Galaxy software (https://usegalaxy.org/). *De novo* genome assembly was performed with Velvet assembler 1.2.10 (3) (considering *k*-mer = 71, cov *k*-mer = 21, and reads >200 bp), and contig annotation was carried out using the Rapid Annotations using Subsystems Technology (RAST) server (4). A draft genome composed of 42 contigs and an *N*_50_ contig of 2,184,772 bp were obtained. The *Halomonas* sp. HG01 genome was found to be 3,665,487 bp long, with 30-fold coverage and a G+C content of 68%. A total of 3,362 coding sequences and 67 RNAs were annotated. Three hundred sixty-seven genes were related to carbohydrate transport and metabolism, with 153 belonging to central carbohydrate metabolism organized as methylglyoxal metabolism (21 genes); pyruvate metabolism II: acetyl-coenzyme A (CoA) and acetogenesis from pyruvate (19 genes); pyruvate-alanine-serine interconversions (12 genes); glyoxylate bypass (6 genes); glycolysis and gluconeogenesis (17 genes); the Entner-Doudoroff pathway (20 genes); dehydrogenase complexes (9 genes); the tricarboxylic acid (TCA) cycle (15 genes); pyruvate metabolism I: anaplerotic reactions and phosphoenolpyruvate (PEP) (15 genes); the pentose phosphate pathway (9 genes); and glycolate-glyoxylate interconversions (9 genes). A deeper study of some of the genes will be performed, emphasizing those genes related to carbohydrate metabolism, P3HB and P3HB-*co*-3HV production, and cell resistance, such as PHB polymerases, PHB depolymerases, and ectoine synthesis. Sequencing of the *Halomonas* sp. HG01 genome and annotation of the specific genes are first efforts at understanding and improving the performance of P3HB and P3HB-*co*-3HV production using different carbon sources and cultured under different conditions.

### Nucleotide sequence accession numbers. 

The contig sequences generated from the whole-genome sequence of *Halomonas* sp. HG01 were deposited in the DDBJ/EMBL/GenBank database under the accession numbers LAEZ00000000. The version described here is the first version, LAEZ01000000.
